# Socioeconomic Inequality in Smoking in Low-Income and Middle-Income Countries: Results from the World Health Survey

**DOI:** 10.1371/journal.pone.0042843

**Published:** 2012-08-29

**Authors:** Ahmad Reza Hosseinpoor, Lucy Anne Parker, Edouard Tursan d'Espaignet, Somnath Chatterji

**Affiliations:** 1 Department of Health Statistics and Information Systems, World Health Organization, Geneva, Switzerland; 2 Department of Public Health, Universidad Miguel Hernández de Elche, Alicante, Spain; 3 CIBER Epidemiología y Salud Pública, Madrid, Spain; 4 Tobacco Free Initiative, World Health Organization, Geneva, Switzerland; Fundación para la Prevención y el Control de las Enfermedades Crónicas No Transmisibles en América Latina (FunPRECAL), Argentina

## Abstract

**Objectives:**

To assess the magnitude and pattern of socioeconomic inequality in current smoking in low and middle income countries.

**Methods:**

We used data from the World Health Survey [WHS] in 48 low-income and middle-income countries to estimate the crude prevalence of current smoking according to household wealth quintile. A Poisson regression model with a robust variance was used to generate the Relative Index of Inequality [RII] according to wealth within each of the countries studied.

**Results:**

In males, smoking was disproportionately prevalent in the poor in the majority of countries. In numerous countries the poorest men were over 2.5 times more likely to smoke than the richest men. Socioeconomic inequality in women was more varied showing patterns of both pro-rich and pro-poor inequality. In 20 countries pro-rich relative socioeconomic inequality was statistically significant: the poorest women had a higher prevalence of smoking compared to the richest women. Conversely, in 9 countries women in the richest population groups had a statistically significant greater risk of smoking compared to the poorest groups.

**Conclusion:**

Both the pattern and magnitude of relative inequality may vary greatly between countries. Prevention measures should address the specific pattern of smoking inequality observed within a population.

## Introduction

There is substantial evidence that poor people in resource rich countries carry the heaviest burden of tobacco related premature death and disability [1]. The association between smoking and poverty is apparent at all levels. Firstly, poor people tend to smoke more both in terms of prevalence and consumption. Additionally, socioeconomic inequality is apparent in initiation: the risk that a young person will begin smoking is greater in less privileged groups [2]; and also in cessation: quit rates are lower in the poorest groups and for those living in socially disadvantaged areas [3]. Furthermore, the risk of dying from smoking is significantly higher in the lowest socioeconomic groups compared to the highest socioeconomic groups [4]. It is apparent that social determinants of smoking may vary between countries [5] and that while health inequalities almost always exist within countries, the magnitude of inequality can vary greatly between countries [6]. Addressing the equity dimensions of tobacco has become an important political and public health priority. Nevertheless, this focus remains to be applied in low and middle income countries where relatively little is known about inequality in tobacco use.

The WHO Framework Convention on Tobacco Control has given an added impetus to implement more effective demand reduction measures, and there has been a considerable reduction in tobacco use in high income countries [7], [8]. To replace these lost consumers, the tobacco industry is using the opportunities offered by globalization to market more aggressively to women and adolescents in low- and middle-income countries [9]–[11], and over 80% of the world's smokers now live in low or middle income countries, where evidence of the relationship between poverty and income levels is lacking relative to high income countries. In this study, we aim (1) to identify the magnitude of inequality in current smoking according to within country wealth status in a large sample of low- and middle-income countries, and (2) to demonstrate the different patterns of wealth-related inequality in these countries. The paper will provide a valuable contribution to the evidence base in this field because of the limited data on disparities within these countries. Such information can have important implications for designing tobacco control interventions.

## Methods

### Study population

The World Health Survey (WHS) was conducted by the World Health Organization in 2002–04 to provide valid, reliable, representative and comparable population data on the health status of adults, aged 18 years and older. The survey was conducted in 70 countries from all regions of the world [12]. All samples were probabilistically selected with every individual being assigned a known non-zero probability of being selected. The samples were nationally representative except in China, Comoros, Congo, Côte d'Ivoire, India, and the Russian Federation, where the WHS was carried out in geographically limited regions. To adjust for the population distribution represented by the UN Statistical Division (http://unstats.un.org/unsd/default.htm) and also non-response, post-stratification corrections were made to sampling weights [13]. Although the WHS collected data on smoking in a couple of high-income countries, this study focuses on 48 low- and middle-income countries surveyed for data on current smoking and demographic and socioeconomic factors.

### Data

A total of 213,807 men and women from the 48 low and middle income countries were available for analyses (Table S1). As most surveys were conducted in 2003, the World Bank's development categories for that year were used to describe each country's income group [14]. Current smoking was defined as a binary variable indicating whether the respondent currently smoked any tobacco product such as cigarettes, cigars or pipes. Current smokers included both daily and occasional smokers. In four countries (India, Bangladesh, Nepal and Myanmar) data were also collected on the use of smokeless tobacco. Although smokeless tobacco is known to cause ill-health, for the purpose of this study individuals who only used smokeless tobacco were considered non-smokers. Wealth was evaluated using a dichotomous hierarchical ordered probit model. This model was used to develop an index of the long-running economic status of households based on ownership of selected assets and use of selected services [15]–[17]. The derived index was divided into five quintiles within each country, where quintile 1 represents the poorest wealth quintile and quintile 5, the richest.

Confounding indicators included participants' sex, age (expressed categorically as 18–29, 30–39, 40–49, 50–59, 60–69 and 70+ years); marital status (married/cohabiting vs. never married vs. divorced/separated/widowed); educational level (no education vs. incomplete primary vs. complete primary vs. secondary/high school vs. college completed or above); employment status (not employed vs. Employed); and area of residence (rural vs. urban).

### Methods of analysis

The overall proportion of current smokers, as well as the proportion by household wealth quintile, was calculated for each country and for men and women separately. These are crude estimations (i.e. not age-standardized) because our main aim was not to compare the smoking prevalence between countries but to compare the magnitude of within country variation in smoking according to wealth. Socioeconomic inequality in smoking prevalence was measured using the relative index of inequality (RII) that takes into account the distribution of smoking as well the distribution of the population across wealth quintiles [18]. A Poisson regression model with a robust variance was used to generate prevalence rate ratio values with 95% confidence intervals. This type of model provides more accurate estimates compared with logit models when the binary outcome has a high prevalence [19]. To calculate RII, individuals were cumulatively ranked (ranging from zero to one) according to their wealth status from highest wealth quintile to lowest. The RII can be interpreted as the prevalence rate ratio between those at top rank (representing the lowest level of wealth) and those at rank zero (representing the highest level of wealth), while taking into the effect across the entire distribution of wealth. Thus, a RII value greater than one indicates that the prevalence of smoking is greater among populations of lower wealth: referred to as *pro-rich inequality*. Conversely, a RII value less than one indicates that smoking is more likely to be prevalent among those with higher wealth level: referred to as *pro-poor inequality* [20]. RII is a relative measure of inequality that is adjusted for variation in overall prevalence across countries. As such, the information on the absolute size of prevalence differences is not reflected in RII [21]. To address this limitation we also provide data on absolute prevalence by wealth quintiles. In addition to reporting unadjusted RII, data were adjusted for age as well as other available confounding factors: marital status, education, employment and urban/rural area. Variables were considered confounders and included in the model, if they influenced the outcome (smoking) and were associated with the main independent variable of the study (wealth).

All analyses were weighted accounting for the individual survey sample designs. The non-independence of observations within the survey clusters were also incorporated in the analysis. Stata 11 was used in all analyses.

### Ethics Statement

Informed consent was obtained in all surveys. A standard consent form approved by the ethics review committee was read to the respondent in the respondent's language. Once the respondent agreed to participate in the survey, if the respondent was literate the form was provided to the respondent to read over and sign and was countersigned by the interviewer. If the respondent was illiterate and gave consent to participate, the interviewer confirmed this consent and signed on the form that the respondent had been read the form, had understood the study and agreed to participate. This procedure was approved by the institutional review boards. The full list of collaborating partners in the 48 countries where the ethical procedure is reviewed and approved is provided in List S1.

## Results

The estimated crude prevalence of current smoking for each of the 48 countries can be found in tables 1 and 2 for males and females respectively, along with estimates for each of the 5 wealth quintiles within these countries. Smoking rates varied widely both by country and sex. The analysis indicated that a higher proportion of men currently smoking compared with women in all of the countries studies. The median smoking prevalences for men and women were 35.9% (95%CI 30.1%–42.0%) and 7.3% (95%CI 4.5%–12.4%), respectively. The current smoking prevalence for men in middle income countries was 46.3% (95%CI 37.0%–53.7%) compared with 26.3% (95%CI 24.0%–34.5%) in low-income countries. Among women, the prevalence rates were 12.5% (95%CI 7.0%–18.2%) for middle-income countries and 5.0% (95%CI 2.7%–7.1%) for low income countries. The lowest overall smoking prevalence for men was found in Ethiopia where 7.4% of men smoked, and the highest was in Latvia where nearly 65% of men smoked. Among women, the lowest prevalence of smoking was observed in Morocco where less than 1% of women were smokers and the highest rates of smoking were seen in Hungary where nearly 40% of women smoked.

**Table 1:** Socioeconomic inequality in smoking prevalence in men in low- and middle-income countries: data from the World Health Survey, 2002–04.

			Across wealth (quintiles)										Relative Index of Inequality (RII)			
Country	Overall		Q1		Q2		Q3		Q4		Q5					
	%	se	%	se	%	se	%	se	%	se	%	Se	Crude RII	(95% CI)	Adjusted RII*	(95% CI)
**Middle-income group**																
Bosnia and Herzegovina	54.2	3.5	71.1	5.5	53.2	7.2	44.1	7.2	50.9	6.6	55.9	7.6	1.16	(0.71–1.90)	1.50	(0.91–2.47)
Brazil	26.9	1.2	38.8	2.5	30.6	2.8	24.1	2.6	22.7	2.5	20.9	2.2	2.20	(1.64–2.95)	1.74	(1.17–2.59)
China	57.5	2.4	72.9	2.8	59.9	3.7	62.4	4.6	53.1	3.3	43.7	5.4	1.70	(1.29–2.23)	1.51	(1.15–1.99)
Croatia	30.6	2.6	38.5	6.9	34.0	6.4	22.6	4.4	29.6	5.9	32.8	6.1	1.08	(0.57–2.05)	1.06	(0.44–2.52)
Czech Republic	37.1	3.9	57.7	9.6	43.7	6.6	41.1	7.6	20.7	5.2	35.0	7.0	2.52	(1.29–4.90)	2.40	(1.16–4.98)
Dominican Republic	17.3	1.2	32.8	3.2	18.5	2.6	16.3	2.5	12.7	2.8	12.1	2.7	3.48	(1.89–6.39)	1.25	(0.59–2.68)
Ecuador	28.7	1.9	37.1	4.6	28.7	3.5	27.2	3.4	23.6	3.1	26.3	3.7	1.62	(1.07–2.46)	1.82	(1.06–3.12)
Estonia	56.9	2.3	62.1	6.7	47.5	5.8	64.8	4.9	61.3	5.9	46.1	6.2	1.14	(0.80–1.62)	1.07	(0.72–1.59)
Georgia	60.9	1.9	50.1	6.0	59.0	4.3	58.3	3.5	63.4	4.0	67.2	2.8	0.75	(0.60–0.93)	0.79	(0.60–1.03)
Hungary	43.7	3.1	82.6	10.1	63.4	8.2	49.4	6.7	34.6	6.0	33.7	5.7	2.70	(1.49–4.89)	2.62	(1.45–4.73)
Kazakhstan	52.0	2.1	56.6	3.2	59.4	3.7	42.3	5.4	57.5	4.0	46.8	3.3	1.24	(1.00–1.52)	1.29	(0.97–1.71)
Latvia	64.7	3.2	67.8	5.5	77.8	6.9	64.9	7.7	61.1	5.9	54.2	6.7	1.44	(1.07–1.95)	1.33	(0.91–1.95)
Malaysia	53.7	1.3	67.4	2.5	60.0	2.7	59.0	2.6	45.1	2.6	39.4	2.5	1.93	(1.65–2.25)	1.38	(1.13–1.68)
Mauritius	42.9	1.5	55.6	3.2	49.4	3.0	45.4	3.1	34.4	2.5	35.1	3.2	1.87	(1.51–2.33)	1.59	(1.25–2.03)
Mexico	36.2	0.8	29.5	1.3	34.8	1.5	38.9	1.4	37.9	1.4	39.0	1.2	0.74	(0.66–0.83)	0.88	(0.78–1.00)
Morocco	32.1	2.2	40.3	4.7	34.0	3.8	38.4	5.4	31.2	4.8	17.5	3.5	2.01	(1.34–3.00)	2.80	(1.44–5.44)
Namibia	29.0	2.0	46.3	4.6	25.2	3.4	25.3	3.5	22.9	3.0	26.1	3.0	2.05	(1.36–3.09)	1.85	(1.16–2.94)
Paraguay	41.5	1.3	62.4	2.6	47.9	2.6	43.8	2.8	28.4	2.5	33.1	2.9	2.42	(1.92–3.06)	2.69	(2.04–3.55)
Philippines	57.8	1.1	67.9	2.2	60.6	2.4	57.3	2.2	55.6	2.2	50.2	2.3	1.41	(1.24–1.60)	1.11	(0.96–1.28)
Russian Federation	57.2	2.7	56.3	4.1	53.2	7.5	58.4	3.9	61.3	5.5	56.5	4.1	0.92	(0.72–1.18)	1.23	(0.99–1.52)
Slovakia	41.6	5.0	33.1	9.6	39.2	12.7	35.0	8.6	52.5	9.2	47.8	9.4	0.61	(0.27–1.36)	0.96	(0.44–2.09)
South Africa	38.7	2.1	43.0	4.7	32.2	3.8	41.3	3.6	40.8	4.2	37.0	5.0	1.02	(0.70–1.48)	0.88	(0.62–1.25)
Sri Lanka	40.2	1.5	56.1	4.5	49.5	3.6	45.5	3.1	38.0	2.3	29.9	3.9	2.14	(1.52–3.02)	2.29	(1.69–3.11)
Swaziland	15.4	2.1	19.9	6.4	10.7	2.8	14.8	4.0	14.3	5.2	16.5	3.9	1.04	(0.37–2.91)	0.94	(0.31–2.81)
Tunisia	53.5	1.4	54.7	3.1	58.6	2.8	56.0	3.1	53.4	2.7	46.2	2.7	1.26	(1.06–1.49)	1.34	(1.06–1.71)
Ukraine	54.4	2.4	55.3	6.0	48.8	4.5	53.8	4.4	57.9	4.0	54.9	4.3	0.92	(0.71–1.19)	1.04	(0.80–1.35)
Uruguay	39.0	1.2	48.6	3.9	42.6	2.7	37.0	3.0	38.4	3.7	32.9	1.4	1.54	(1.30–1.82)	1.51	(1.20–1.90)
**Low-income group**																
Bangladesh	56.1	1.4	72.2	2.9	64.1	2.9	56.7	2.8	48.6	2.7	44.2	2.6	1.87	(1.60–2.18)	1.40	(1.14–1.72)
Burkina Faso	24.3	1.6	29.9	3.4	25.8	2.8	21.6	2.5	17.5	2.5	26.2	3.5	1.53	(1.01–2.33)	2.02	(1.20–3.40)
Chad	18.3	1.9	22.8	4.0	19.9	3.2	19.3	3.2	18.2	2.3	14.8	2.0	1.61	(1.00–2.59)	1.62	(0.99–2.67)
Comoros	35.6	3.1	39.0	8.0	37.9	6.5	31.6	4.6	39.5	6.0	32.4	5.5	1.18	(0.66–2.12)	1.23	(0.60–2.50)
Congo	17.3	2.4	31.2	5.8	28.6	4.8	17.7	4.5	10.7	3.4	9.9	4.2	5.08	(1.70–15.16)	1.58	(0.48–5.22)
Cote d'Ivoire	20.9	1.4	28.0	3.0	21.3	2.8	22.0	2.8	19.5	2.8	18.0	3.1	1.58	(0.98–2.54)	1.32	(0.77–2.25)
Ethiopia †	7.4	1.1	5.3	2.0	9.5	2.2	8.1	1.8	8.8	1.4	4.8	1.2	1.22	(0.55–2.70)	-	-
Ghana	10.6	1.0	21.5	3.0	12.7	1.9	9.6	1.8	6.1	1.3	8.4	1.8	3.56	(1.79–7.09)	2.27	(1.20–4.31)
India	35.3	1.6	46.7	3.2	45.8	2.9	37.8	3.8	23.5	2.9	21.8	3.1	2.78	(1.85–4.16)	1.62	(1.07–2.46)
Kenya	26.9	2.4	33.1	5.4	26.9	4.0	25.2	3.9	25.6	4.0	26.7	5.5	1.17	(0.64–2.13)	1.00	(0.48–2.10)
Lao People's Democratic Republic	63.4	1.6	77.1	2.9	72.7	2.7	61.0	3.2	62.7	2.9	41.5	2.8	1.89	(1.62–2.20)	1.68	(1.40–2.03)
Malawi	25.6	1.7	40.9	3.2	34.9	2.7	24.0	3.3	15.9	2.4	13.3	2.7	4.46	(3.10–6.43)	3.03	(2.04–4.50)
Mali	25.7	1.4	27.3	3.2	28.2	3.2	24.7	2.7	24.2	2.5	25.9	3.1	1.09	(0.73–1.63)	1.38	(0.92–2.06)
Mauritania	31.2	2.2	27.8	4.3	22.3	4.3	23.4	4.0	30.6	4.2	38.6	4.0	0.50	(0.32–0.80)	1.03	(0.58–1.84)
Myanmar	47.5	1.7	52.5	3.9	53.7	3.2	46.0	2.8	48.4	2.5	40.3	2.5	1.38	(1.12–1.70)	1.40	(1.13–1.72)
Nepal	33.5	1.2	43.7	2.6	36.8	2.5	36.0	2.2	30.4	2.2	26.0	2.0	1.87	(1.50–2.34)	1.21	(0.92–1.58)
Pakistan	33.1	1.4	40.5	2.5	35.4	2.5	35.6	2.7	32.0	2.6	19.1	2.1	1.90	(1.52–2.37)	1.40	(1.08–1.82)
Senegal	25.4	2.1	28.9	4.6	25.5	4.7	24.4	4.0	21.2	4.1	26.3	3.8	1.16	(0.66–2.04)	1.72	(0.87–3.38)
Viet Nam	51.4	2.8	66.9	3.5	59.8	4.3	43.0	5.6	45.0	4.2	46.7	4.7	1.63	(1.25–2.14)	1.07	(0.81–1.41)
Zambia	23.8	1.2	36.8	3.5	27.5	2.6	21.5	2.5	22.9	2.7	13.0	2.1	2.81	(1.95–4.04)	3.22	(1.90–5.45)
Zimbabwe	26.3	1.6	37.3	4.5	29.6	3.9	26.4	3.7	20.0	2.8	23.5	2.8	1.84	(1.25–2.70)	1.61	(0.97–2.67)

* Adjustments were made for age, marital status, education, employment and rural/urban residence.

† Adjusted RII could not be obtained due to the reduced number of smokers within some of the categories of the confounding variables.

**Table 2:** Socioeconomic inequality in smoking prevalence in women in low- and middle-income countries: data from the World Health Survey, 2002–04.

			Across wealth (quintiles)										Relative Index of Inequality (RII)			
Country	Overall		Q1		Q2		Q3		Q4		Q5					
	%	se	%	se	%	se	%	se	%	se	%	Se	Crude RII	(95% CI)	Adjusted RII*	(95% CI)
**Middle-income group**																
Bosnia and Herzegovina	34.0	5.0	13.7	4.2	40.0	9.7	33.9	8.1	36.8	7.5	37.7	6.2	0.63	(0.31–1.32)	1.20	(0.55–2.66)
Brazil	17.8	0.9	24.2	2.2	18.6	1.8	18.6	1.9	15.5	1.9	13.8	1.8	1.95	(1.37–2.75)	1.59	(1.01–2.50)
China	3.4	0.6	5.4	2.0	5.4	1.7	2.9	1.4	2.7	0.9	1.8	0.7	3.94	(1.44–10.76)	1.60	(0.53–4.86)
Croatia	23.5	2.2	11.4	3.6	24.0	4.4	19.8	4.3	29.4	5.2	27.1	4.6	0.51	(0.28–0.91)	0.86	(0.37–2.02)
Czech Republic	25.5	3.1	20.1	5.4	37.1	7.0	19.5	5.6	27.7	6.3	22.1	7.2	1.03	(0.49–2.15)	1.73	(0.62–4.88)
Dominican Republic	12.5	1.0	24.5	2.9	19.0	2.5	16.8	2.6	10.7	1.9	5.2	1.6	6.44	(3.24–12.77)	6.61	(3.20–13.65)
Ecuador	7.1	0.9	4.2	1.3	5.5	1.6	5.8	1.3	8.3	2.0	11.8	2.5	0.28	(0.12–0.63)	0.44	(0.19–1.03)
Estonia	25.1	1.6	22.7	3.8	21.5	3.1	29.1	2.9	30.2	5.0	22.5	3.9	0.85	(0.48–1.49)	2.22	(1.17–4.20)
Georgia	6.4	1.3	1.9	1.1	2.4	1.1	4.8	1.8	8.2	2.5	11.6	2.6	0.11	(0.03–0.37)	0.72	(0.16–3.20)
Hungary	39.5	3.0	65.6	13.1	44.5	7.8	44.2	6.4	38.9	5.4	30.3	5.5	2.00	(1.12–3.57)	1.68	(0.87–3.25)
Kazakhstan	9.6	1.9	2.7	0.9	6.5	1.4	8.2	2.0	16.5	4.6	14.7	3.8	0.15	(0.06–0.38)	0.26	(0.09–0.76)
Latvia	24.0	2.1	22.7	4.3	26.5	5.2	29.4	5.1	22.0	4.3	18.1	4.0	1.33	(0.80–2.21)	2.12	(1.14–3.95)
Malaysia	2.6	0.4	5.9	1.5	1.4	0.5	2.8	0.8	2.6	0.8	1.2	0.5	4.18	(1.37–12.78)	3.18	(1.13–8.95)
Mauritius	2.8	0.5	2.5	0.7	2.9	0.8	2.4	0.8	2.1	0.7	4.1	1.3	0.65	(0.19–2.22)	0.96	(0.23–3.93)
Mexico	15.2	0.6	7.0	0.7	12.0	0.8	13.9	0.8	17.4	1.1	22.9	1.0	0.28	(0.23–0.34)	0.40	(0.31–0.51)
Morocco †	0.2	0.1	0.0	0.0	0.4	0.3	0.0	0.0	0.4	0.3	0.0	0.0	1.00	(0.06–16.16)	-	-
Namibia	12.4	1.2	17.8	2.5	12.3	2.2	10.1	2.0	7.6	1.9	12.4	2.6	2.02	(1.05–3.87)	0.98	(0.46–2.10)
Paraguay	13.3	0.8	17.9	1.9	16.3	1.9	14.3	1.8	9.7	1.3	12.2	1.5	1.74	(1.13–2.70)	1.53	(0.88–2.64)
Philippines	12.5	0.7	17.2	1.7	14.4	1.4	12.1	1.2	12.1	1.3	8.8	1.3	2.15	(1.46–3.18)	1.79	(1.19–2.70)
Russian Federation	11.2	1.2	9.6	2.2	8.8	1.9	8.7	1.7	12.5	2.1	17.1	4.1	0.45	(0.19–1.04)	1.14	(0.45–2.89)
Slovakia	24.2	3.0	13.4	4.1	25.7	8.0	24.0	5.1	27.7	6.3	35.3	7.3	0.37	(0.16–0.84)	0.80	(0.35–1.80)
South Africa	12.5	1.3	7.6	2.0	9.4	2.2	14.0	3.0	18.0	3.4	15.1	2.7	0.36	(0.19–0.69)	0.21	(0.09–0.48)
Sri Lanka	3.0	0.6	6.4	1.9	4.7	1.5	3.0	1.0	1.7	0.6	2.4	1.0	3.56	(0.79–16.10)	2.70	(0.84–8.71)
Swaziland	3.2	0.9	8.8	3.4	1.7	0.9	0.2	0.2	4.1	1.6	2.3	2.3	4.12	(0.28–61.63)	4.00	(0.73–22.00)
Tunisia	2.2	0.3	3.4	0.8	0.7	0.4	2.3	0.8	2.3	0.7	2.5	0.8	0.97	(0.33–2.84)	2.20	(0.52–9.26)
Ukraine	10.7	1.2	7.7	1.8	6.1	1.4	12.0	2.3	13.2	3.0	14.0	2.2	0.40	(0.21–0.74)	0.96	(0.49–1.85)
Uruguay	28.8	1.9	28.2	5.5	30.8	3.3	26.3	3.3	27.3	1.8	30.9	1.3	0.94	(0.66–1.33)	1.24	(0.88–1.75)
**Low-income group**																
Bangladesh	6.5	0.8	8.2	1.5	6.0	1.2	8.3	1.5	5.9	1.6	3.5	0.9	2.02	(1.13–3.61)	1.02	(0.49–2.09)
Burkina Faso †	11.5	1.6	12.6	2.4	14.1	2.4	10.9	2.1	10.5	2.2	8.2	2.9	1.62	(0.87–3.03)	-	-
Chad	3.3	1.0	1.6	0.7	5.1	2.0	2.7	1.1	3.8	2.0	3.6	1.2	0.68	(0.29–1.61)	0.34	(0.10–1.23)
Comoros †	22.2	3.8	38.3	8.7	14.7	5.5	21.1	6.8	16.3	5.9	20.2	8.7	2.33	(0.84–6.46)	-	-
Congo †	1.9	0.6	4.9	2.5	0.9	0.5	2.9	1.8	1.4	0.7	0.3	0.3	10.21	(1.60–65.08)	-	-
Cote d'Ivoire	2.9	0.5	3.4	1.6	5.1	1.8	3.9	1.3	1.8	0.8	1.1	0.6	4.97	(1.46–16.95)	10.00	(2.24–44.67)
Ethiopia †	0.5	0.2	0.4	0.4	1.2	0.6	0.7	0.4	0.6	0.4	0.1	0.1	3.55	(0.58–21.54)	-	-
Ghana	1.3	0.3	2.6	0.7	1.4	0.7	0.9	0.7	0.7	0.4	1.3	0.5	2.43	(0.53–11.21)	1.95	(0.49–7.85)
India	7.6	1.1	12.4	3.7	8.6	1.8	8.4	1.7	4.3	1.1	3.1	1.0	4.55	(1.98–10.48)	3.80	(0.94–15.39)
Kenya	2.0	0.6	3.2	0.9	3.3	1.0	3.8	2.3	0.8	0.4	0.2	0.2	13.08	(4.35–39.33)	4.77	(1.21–18.76)
Lao People's Democratic Republic †	13.0	1.4	28.3	3.6	17.8	2.4	12.7	2.1	5.0	1.3	1.8	0.8	17.38	(9.76–30.94)	-	-
Malawi †	5.7	0.9	9.5	1.5	7.5	1.6	6.8	1.5	3.5	1.1	0.7	0.5	7.48	(3.93–14.22)	-	-
Mali †	3.0	0.6	4.0	1.6	3.8	1.6	3.5	1.3	2.9	1.1	0.5	0.5	3.77	(1.12–12.67)	-	-
Mauritania	5.0	0.9	2.8	1.1	1.6	0.8	5.6	2.2	7.2	1.8	5.8	1.5	0.37	(0.14–0.98)	1.43	(0.31–6.61)
Myanmar	12.4	1.1	21.8	2.5	18.2	2.3	12.5	1.5	8.5	1.1	4.6	1.0	6.48	(4.11–10.23)	3.76	(2.36–6.00)
Nepal	19.5	1.0	28.5	2.1	25.6	2.0	18.7	1.5	17.9	1.8	9.7	1.3	3.41	(2.50–4.65)	2.19	(1.58–3.03)
Pakistan	6.4	0.7	7.4	1.5	6.8	1.3	7.4	1.8	6.3	1.6	3.8	1.2	1.71	(0.86–3.39)	1.35	(0.61–2.98)
Senegal †	1.7	0.6	4.7	2.0	0.0	0.0	0.4	0.4	0.5	0.4	1.9	1.4	8.62	(0.25–297.91)	-	-
Viet Nam	2.5	0.5	3.2	1.2	2.1	0.8	2.2	0.8	3.4	1.4	1.8	1.2	1.43	(0.30–6.76)	0.59	(0.10–3.40)
Zambia	5.9	0.8	11.5	1.6	7.9	2.3	2.6	1.0	3.4	1.2	3.9	1.5	5.95	(2.45–14.45)	1.68	(0.55–5.07)
Zimbabwe †	3.1	0.5	6.4	2.0	3.6	1.0	3.1	0.8	1.9	0.7	1.7	0.8	5.51	(1.49–20.38)	-	-

* Adjustments were made for age, marital status, education, employment and rural/urban residence.

† Adjusted RII could not be obtained due to the reduced number of smokers within some of the categories of the confounding variables.

### Smoking Prevalence by Within Country Wealth Status among Men and Women

There was great variation in smoking within countries with respect to wealth, and in some cases extremely high rates were observed in certain groups. The smoking prevalence was above 70% in poorest men from Bangladesh, Bosnia and Herzegovina, China and Lao People's Democratic Republic, and in Hungary this figure was over 80%. Similarly, the prevalence of smoking in the poorest women in Hungary was among the highest observed in women (over 65%). High rates of smoking were not always confined to the poorest groups. For example, in Bosnia and Herzegovina, Georgia, Latvia, Philippines, Russian Federation and Ukraine, even in the richest population group smoking in men was more common than not (i.e. the prevalence was above 50%).

### Within-country Relative Inequalities in Smoking by Wealth Status among Men - Unadjusted Results

**Figure 1:**
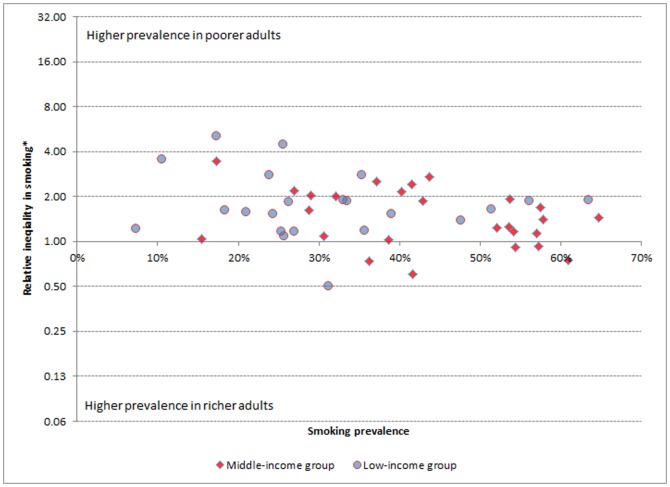
Prevalence of smoking versus socioeconomic inequality in smoking in men from 48 low- and middle-income countries (World Health Survey, 2002–2004). * Relative index of inequality according to wealth.

With regard to socioeconomic inequality in males, smoking was disproportionately prevalent in the poor in the majority of countries. In a number of the countries studied, the poorest men were over two and a half times more likely to smoke than the richest men (table 1). Nevertheless, it is also apparent that in three countries (Georgia, Mauritania and Mexico) there was significant pro-poor inequality for males; that is men in more affluent groups smoked more than the poorest groups. Figure 1 plots the prevalence of smoking against the relative inequality in smoking in men. It is apparent that the majority of countries appear above the central axis reflecting pro-rich inequality in smoking with a higher prevalence in poorer men. Visually, the relative inequalities do not seem to vary according to country income group. In line with this, the median RII was more or less similar in low-income and middle-income groups (1.63 vs. 1.44 respectively).

### Within-country Relative Inequalities in Smoking by Wealth Status among Women -Unadjusted Results

**Figure 2:**
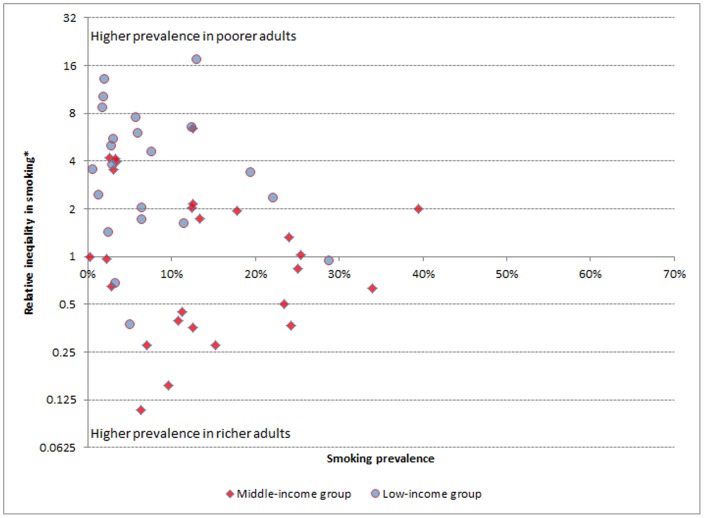
Prevalence of smoking versus socioeconomic inequality in smoking in women from 48 low- and middle-income countries (World Health Survey, 2002–2004). * Relative index of inequality according to wealth.

Socioeconomic inequality in women was much more mixed, showing types of both pro-rich and pro-poor inequalities. Pro-rich socioeconomic inequality was most extreme in Congo, Kenya and Lao People's Democratic Republic where the poorest women had more than 10 times more risk of smoking compared to the richest women. Conversely, in Ecuador, Georgia, Kazakhstan and Mexico women in the richest women in the population had over three and a half times the risk of smoking compared to the poorest groups. In fact, this type of pro-poor inequality in women was observed in 16 of the countries studied, and was statistically significant among 9 (table 2). There are remarkable differences between figures 1 and 2 which show the prevalence of smoking against the relative socioeconomic inequality in smoking in men and women respectively. In the latter, a large number of countries appear in the lower part of the figure, below the central axis, reflecting the fact that smoking is more prevalent in richer women in these countries. Moreover, a number of these cases of pro-poor inequality belong to middle income countries. This is reflected in the fact that the median RII in middle income countries is 0.97 (pro-rich and pro-poor inequalities have cancelled each other out), while in low-income countries, the majority of which demonstrated pro-rich inequality, the median RII was 3.77.

### Relative Inequalities in Smoking by Wealth Status among Men and Women— Adjusted Results

After controlling for age, marital status, education, employment and urban/rural residence, pro-rich socioeconomic inequality in smoking was still statistically significant among men in nearly half of the countries studies. In nine countries from both low- and middle-income groups the poorest men were at least two times more likely to smoke than the richest ones even after controlling for these factors (table 1). The pro-poor inequality that was apparent in Georgia was not statistically significant after controlling for confounders. Statistically significant pro-rich inequality was seen among women living in 10 out of 38 countries where adjustment for confounders could be done (table 2). Kazakhstan, Mexico and South Africa showed statistically significant pro-poor inequality where the richest women were at least two times and half more likely to smoke than the poorest ones, after adjusting for age, education, marital status, employment and urban/rural residence.

## Discussion

This study used data from 48 countries that took part in the world Health Surveys to analyse socioeconomic inequality in current daily or occasional tobacco smoking in men and women from low- or middle-income countries. We have shown that the magnitude and direction of socioeconomic inequality varies substantially between counties. It is conventional wisdom that smoking levels are highest in the poorest groups of the population, but this is not always the case as shown in our study. Particularly in women and in middle income countries, we observed a significant pattern of pro-poor inequality – risk of smoking was higher in the wealthiest populations groups. More research is required in order to see if this pattern persists over time, and to explore the situation in other middle-income countries that are not included in this study. Interestingly, pro-poor inequality in smoking according to education has also been observed in women in Southern Europe [6].

Apart from these examples of pro-poor inequality, we have shown that many countries exhibit pro-rich smoking inequality. This type of inequality is especially relevant because smoking in the poorest population groups can have additional effects that go beyond the direct health effects, further exacerbating health-related inequality. It has been shown that in low socioeconomic groups, already scarce income may be diverted away from things such as education, health care, housing, or quality food, in order to purchase cigarettes [22]. Poor rural households in China are reported to spend over 10% of their total household expenditures on cigarettes [23], which could have indirect effect on other family members. For example, paternal smoking in poor families in Indonesia has been shown to divert spending from fruit and vegetables and exacerbate child malnutrition [24] and increase under-five mortality [25].

An individual's risk of smoking is likely to be influenced by a number of linked social factors as well as their cumulative effect over the life course. Here we assessed inequality by wealth, but it is clear that other factors such as education are also key determinants of inequality. Nevertheless, after we controlled for other factors such as age, marital status, education, rural/urban dwelling and employment, we showed that wealth remains a significant determinant of smoking risk in many countries. One policy implication of this finding is that interventions to reduce the burden of tobacco related ill health should also be directed at poverty itself and its associates. Similarly, some authors have suggested that anti-smoking policies should focus on improving the standard of living among smokers [26]. Furthermore, the data presented in this paper point to a major equity challenge in the development of tobacco control and it is suggested that, in addition to strong country commitment to implementing the measures of the FCTC, monitoring the implementation of the convention should include an equity lens that pays special attention to the possibility that some population groups may be better positioned to access, utilize and derive the health benefits from tobacco control interventions. For example, although cessation services in the UK were able to reach the lower socioeconomic groups, the levels of cessation among these groups was much lower than the highest socioeconomic group [27].

Apart from estimating the magnitude of inequality, it is also important to consider the pattern of inequality within the country. Different patterns of inequality have been described in childhood malnutrition [28] and similarly, the pattern of inequality in smoking may have different implications for tobacco control policies. For example, in the current study, the socioeconomic distribution of smoking among men in Namibia, and among women in Comoros, followed a pattern which can be considered as “exclusion”: the prevalence of smoking is fairly similar in most population groups but was almost double that in the poorest group representing a small deprived minority. However, the data revealed quite a different pattern among men in Morocco which could parallel the “mass deprivation” pattern of inequality: while in most wealth groups over 30% of men smoked, the richest group represented a small privileged minority with a much lower smoking prevalence. With regard to prevention and control, we can draw on the ideas of Geoffrey Rose and the strategies of preventative medicine [29]. Population based strategies aimed at reducing tobacco use across the whole population would be important in countries with the “mass deprivation” pattern of inequality, as well as countries with little or no inequality but high smoking prevalence. On the other hand, interventions targeted at vulnerable high-risk groups may be more appropriate for countries with other patterns of inequality such as “exclusion”. In other populations, such as men in Zambia, or women in Mexico, a more linear social gradient was observed. With each step in wealth, a progressively lower rate of smoking was observed. In these populations a combination of population based prevention, and high-risk strategies would be necessary.

Inequality in tobacco use translates into inequality in tobacco-related premature death and disease. In this regard, the disparities between the wealthiest members of society and the poorest members should be considered a health inequity, because they are unfair and preventable [30]. Tobacco control strategies do exist, and so it is our social obligation to reduce tobacco-related health inequity. In this study we have highlighted the problem of socioeconomic inequality in smoking in low and middle income countries where evidence is scarce and limited resources for health, poorer prevention programs and control strategies can lead to an even greater disease burden. The message is a timely one because it comes as the international community begins to shift its focus in the developing world, from infectious diseases towards cancer and other non-communicable diseases. Prevention measures should be designed and implemented to address the specific pattern of smoking inequality observed within a population, in order to close the gap between rich and poor.

## Supporting Information

Table S1 **Study population (final unweighted sample count) by country and sex, World Health Survey, 2002–2004.**
(DOC)

List S1 **List of in-country collaborating partners.**
(XLS)
