# Supplementary material for: Socioeconomic Inequality in Smoking in Low-Income and Middle-Income Countries: Results from the World Health Survey
Source: PLoS One. 2012 Aug 29;7(8):e42843. doi: 10.1371/journal.pone.0042843 (PMC3430638; doi:10.1371/journal.pone.0042843)
Supplement: Table S1 — Study population (final unweighted sample count) by country and sex, World Health Survey, 2002–2004. (DOC) [file pone.0042843.s001.doc]

**Table S1** Study population (final unweighted sample count) by country and sex, World Health Survey, 2002-2004

| **Middle income*** | | | |  | **Low income*** | | | |
| --- | --- | --- | --- | --- | --- | --- | --- | --- |
| **Country** | **Males** | **Females** | **Total** |  | **Country** | **Males** | **Females** | **Total** |
|  |  |  |  |  |  |  |  |  |
| Bosnia and Herzegovina | 429 | 586 | 1,015 |  | Bangladesh | 2,531 | 2,908 | 5,439 |
| Brazil | 2,110 | 2,691 | 4,801 |  | Burkina Faso | 2,223 | 2,459 | 4,682 |
| China | 1,941 | 2,008 | 3,949 |  | Chad | 2,049 | 2,165 | 4,214 |
| Croatia | 384 | 557 | 941 |  | Comoros | 440 | 247 | 687 |
| Czech Republic | 407 | 499 | 906 |  | Congo | 874 | 1,015 | 1,889 |
| Dominican Republic | 2,051 | 2,363 | 4,414 |  | Cote d'Ivoire | 1,653 | 1,188 | 2,841 |
| Ecuador | 1,652 | 2,013 | 3,665 |  | Ethiopia | 2,315 | 2,477 | 4,792 |
| Estonia | 356 | 638 | 994 |  | Ghana | 1,623 | 1,959 | 3,582 |
| Georgia | 1,114 | 1,505 | 2,619 |  | India | 4,465 | 4,752 | 9,233 |
| Hungary | 281 | 302 | 583 |  | Kenya | 1,813 | 2,450 | 4,263 |
| Kazakhstan | 1,537 | 2,929 | 4,466 |  | Lao People's Democratic Republic | 1,858 | 2,048 | 3,906 |
| Latvia | 284 | 564 | 848 |  | Malawi | 2,142 | 2,895 | 5,037 |
| Malaysia | 2,568 | 3,079 | 5,647 |  | Mali | 1,761 | 1,059 | 2,820 |
| Mauritius | 1,833 | 1,974 | 3,807 |  | Mauritania | 1,150 | 1,830 | 2,980 |
| Mexico | 16,249 | 22,078 | 38,327 |  | Myanmar | 2,549 | 3,331 | 5,880 |
| Morocco | 1,937 | 2,120 | 4,057 |  | Nepal | 3,300 | 4,400 | 7,703 |
| Namibia | 1,489 | 2,196 | 3,685 |  | Pakistan | 3,287 | 2,574 | 5,861 |
| Paraguay | 2,325 | 2,752 | 5,077 |  | Senegal | 899 | 646 | 1,545 |
| Philippines | 4,613 | 5,352 | 9,965 |  | Viet Nam | 1,554 | 1,890 | 3,444 |
| Russian Federation | 1,541 | 2,748 | 4,289 |  | Zambia | 1,706 | 2,032 | 3,738 |
| Slovakia | 567 | 1,099 | 1,666 |  | Zimbabwe | 1,425 | 2,430 | 3,855 |
| South Africa | 1,025 | 1,105 | 2,130 |  |  |  |  |  |
| Sri Lanka | 2,806 | 2,926 | 5,732 |  |  |  |  |  |
| Swaziland | 725 | 986 | 1,711 |  |  |  |  |  |
| Tunisia | 2,284 | 2,609 | 4,893 |  |  |  |  |  |
| Ukraine | 828 | 1,467 | 2,295 |  |  |  |  |  |
| Uruguay | 1,436 | 1,498 | 2,934 |  |  |  |  |  |
|  |  |  |  |  |  |  |  |  |
| **Total** | **54,772** | **70,644** | **125,416** |  | **Total** | **41,627** | **46,764** | **88,391** |

**World Development Report 2005*
